# Sinus Lift With Simultaneous Mucous Retention Cyst Drainage for Implant Rehabilitation: Clinical, Tomographic, and Histologic Assessments in a Six-Year Follow-Up Case Report

**DOI:** 10.7759/cureus.99624

**Published:** 2025-12-19

**Authors:** Erick R Silva, Thales Bianchi, Letícia G Artioli, Ailton C Moraes Filho, Daniele Botticelli, Samuel P Xavier

**Affiliations:** 1 Department of Oral and Maxillofacial Surgery and Periodontology, Faculty of Dentistry of Ribeirão Preto, University of São Paulo, Ribeirão Preto, BRA; 2 Department of Oral Implantology, ARDEC Academy, Rimini, ITA

**Keywords:** cbct densitometry, delayed implant placement, guided bone regeneration, histomorphometry, lateral window, maxillary sinus floor elevation, mucous retention cyst, xenograft

## Abstract

We aim to report the long-term clinical, tomographic, and histologic outcomes of simultaneous drainage of a maxillary mucous retention cyst (MRC) and lateral sinus floor elevation (SFE), followed by delayed guided implant placement, with a six-year follow-up. A 47-year-old male with an atrophic posterior maxilla (residual bone height (RBH): 1.0 mm) presented with a dome-shaped MRC measuring 22.13 × 15.86 × 27.94 mm (3,266.83 mm³). A lateral window SFE was performed with cyst drainage, careful elevation of the Schneiderian membrane, and grafting using particulate xenograft, covered by a collagen membrane. Cone-beam computed tomography (CBCT) was obtained at one month (T1), eight months (T2), and six years (T3) to assess graft volume and density. At eight months, a guided Ø4.3 × 8.5 mm implant was placed, and a trephine core biopsy was collected for histologic and histomorphometric evaluation. Postoperative healing was uneventful, and the patient remained asymptomatic throughout follow-up. At T1, graft volume was 1,978.61 mm³ (297.24 HU), and the cyst regressed by 6.10% compared with baseline. At T2, graft volume decreased to 1,737.67 mm³ (-12.18% vs. T1), with increased density (690.74 HU), and the MRC was radiographically resolved. Histomorphometric analysis revealed 25.76% new bone, 47.20% residual graft, and 27.04% connective tissue. At T3, graft volume remained 1,508.57 mm³ (-23.76% vs. T1), with a density of 785.91 HU, indicating long-term bone maturation and consolidation. The maxillary sinus remained functional and asymptomatic, with stable peri-implant tissues and no recurrence of the MRC. Simultaneous management of an MRC during lateral SFE allowed safe cyst drainage, predictable graft consolidation, histologic confirmation of new bone formation, and long-term implant success. This single-stage approach represents a pragmatic and effective treatment option in carefully selected cases of atrophic maxilla with cystic sinus lesions.

## Introduction

The rehabilitation of the atrophic posterior maxilla with dental implants often presents substantial anatomical and biological challenges. These challenges mainly arise from reduced residual bone height (RBH), poor bone quality, and the close anatomical relationship with the maxillary sinus, which may compromise implant placement and primary stability [1,2]. To overcome these limitations, sinus floor elevation (SFE) procedures - particularly the lateral window approach - have become a cornerstone of pre-prosthetic surgery, consistently providing predictable vertical bone augmentation [3,4].

The maxillary sinus is prone to several pathologies, with mucous retention cysts (MRCs) among the most common incidental findings on panoramic radiographs and cone-beam computed tomography (CBCT) scans [5]. Their reported prevalence ranges between 1.6% and 13.9% in the general population [6,7]. These dome-shaped, fluid-density lesions typically result from obstruction of seromucous glands within the Schneiderian membrane and are usually asymptomatic, being detected incidentally during implant planning [5,8].

Although generally considered benign, the presence of an MRC adds complexity to sinus augmentation procedures. Concerns include a higher risk of membrane perforation, potential contamination of graft material, and compromised graft and implant outcomes [9-11]. Traditionally, management strategies ranged from conservative observation to staged surgical approaches, such as cyst enucleation followed by delayed SFE [12].

More recently, a single-stage strategy has gained attention, whereby the MRC is drained or enucleated simultaneously with lateral SFE. Retrospective analyses and case series have reported encouraging clinical and histologic outcomes using this combined approach, especially in cases of larger lesions [13-16]. Nonetheless, long-term data combining clinical, radiographic, and histologic assessments, particularly in conjunction with guided implant placement, remain scarce.

This report presents a six-year clinical, tomographic, and histologic follow-up of a case managed with single-stage cyst drainage and lateral SFE, followed by delayed guided implant placement.

## Case presentation

Patient information and ethics

A 47-year-old systemically healthy male patient presented to the School of Dentistry of Ribeirão Preto, University of São Paulo, Ribeirão Preto, Brazil, seeking prosthetic rehabilitation for missing teeth in the posterior maxilla. His chief complaint was the functional and esthetic deficit caused by the edentulous area. The patient reported no relevant medical history, was not taking any medications, had no allergies, and was a non-smoker with no regular alcohol consumption.

**Figure 1:**
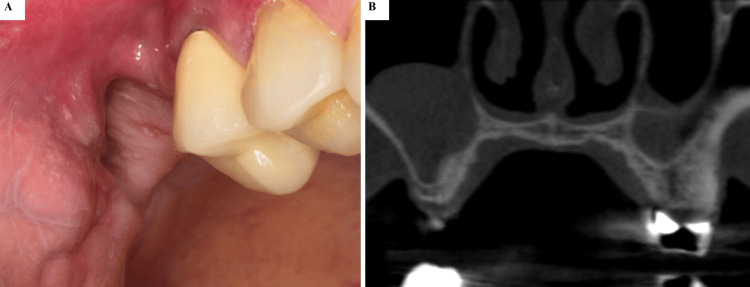
(A-B) Preoperative Intraoral and Tomographic View Preoperative intraoral view showing the atrophic alveolar ridge in the right posterior maxilla (sites #16 and #17), with no clinical signs of inflammation or mucosal alterations.

Clinical examination revealed the absence of teeth #16 and #17 and an atrophic alveolar ridge. There were no clinical signs of inflammation, pain, or sinus-related symptoms (Figure 1). The adjacent maxillary premolar was clinically and radiographically evaluated and exhibited no signs of periapical pathology, endodontic disease, or periodontal compromise. During the preoperative radiographic assessment, a soft-tissue density lesion arising from the sinus floor was incidentally identified. The radiographic appearance was consistent with a presumed MRC, characterized by its well-defined, dome-shaped morphology, absence of cortical disruption, and typical sinus floor origin. As no solid tissue was available for histopathological analysis, this represents a provisional radiologic diagnosis. Differential considerations include antral pseudocyst, postoperative maxillary cyst, mucocele, benign odontogenic cysts extending into the sinus, and inflammatory polyps.

Preoperative assessment (T0)

**Figure 2:**
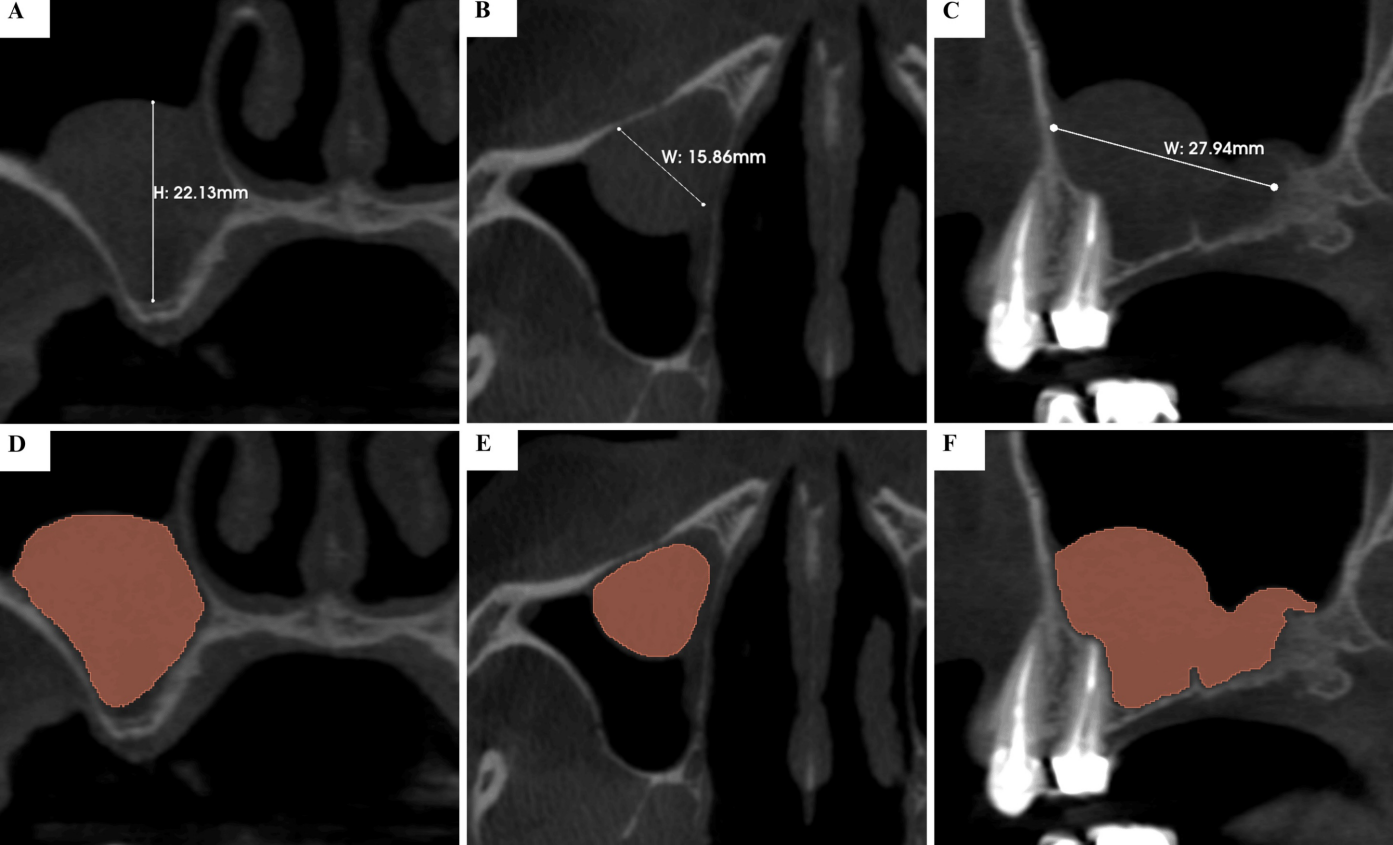
Preoperative Cone-Beam Computed Tomography (CBCT) of the Right Maxillary Sinus Showing a Mucous Retention Cyst Preoperative CBCT at baseline (T0) showing a probable mucous retention cyst, well-defined and dome-shaped, in the right maxillary sinus. The lesion measured 22.13 mm in height (A), 15.86 mm in bucco-palatal width (B), and 27.94 mm in mesio-distal width (C), occupying a substantial portion of the sinus and closely related to the planned implant site in the edentulous posterior maxilla. The brown region of interest (ROI) highlighted in panels (D)-(F) corresponds to the area of the probable mucous retention cyst within the right maxillary sinus.

Baseline CBCT, acquired in July 2019, revealed a severely atrophic alveolar ridge with an RBH of 1.0 mm below the sinus floor. A dome-shaped lesion, consistent with an MRC, was observed in the right maxillary sinus. The cyst measured 22.13 mm in height (Figure 2A), 15.86 mm in bucco-palatal width (Figure 2B), and 27.94 mm in mesio-distal width (Figure 2C), corresponding to a calculated volume of 3,266.83 mm³. The lesion occupied a large portion of the sinus and was closely related to the planned implant site (Figures 2D-2F).

Treatment planning

Given the limited RBH (1.0 mm) and the large MRC, SFE was essential for rehabilitation. In line with contemporary approaches aimed at reducing treatment time and morbidity - and considering the asymptomatic nature of the cyst - a single-stage strategy was selected: drainage of the MRC simultaneously with a lateral window SFE, followed by delayed implant placement.

Regarding implant selection, the decision to place an 8.5-mm implant, rather than a longer fixture, was based on prosthetically driven planning combined with three-dimensional evaluation of bone distribution. Adequate primary stability and circumferential bone support were achieved without the need for greater apical extension into the augmented sinus.

Surgical procedure

**Figure 3:**
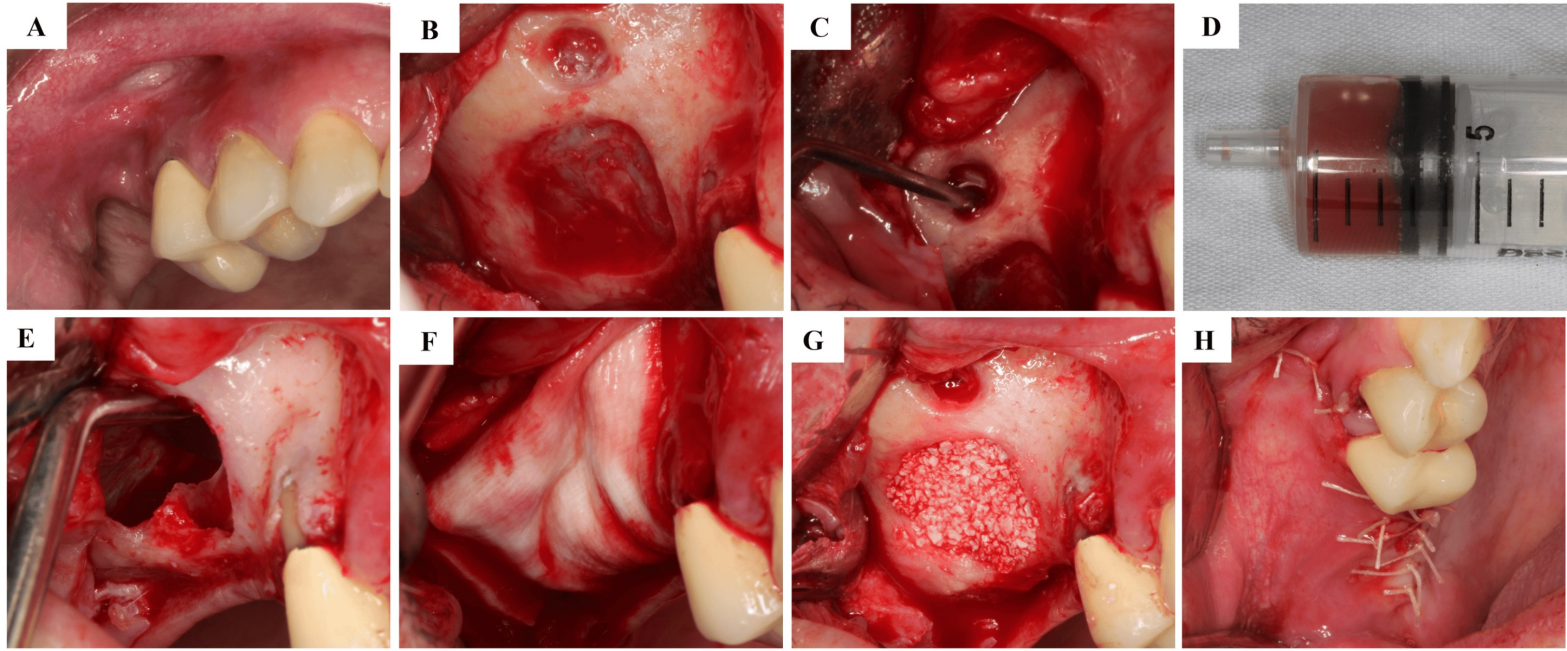
Intraoperative Sequence of Lateral Sinus Floor Elevation With Cyst Drainage Intraoperative sequence of the lateral window sinus floor elevation with simultaneous cyst drainage. (A) Flap elevation and exposure of the lateral sinus wall; (B) Lateral osteotomy; (C) Exposure of the mucous retention cyst; (D) Aspiration of cystic contents; (E) Schneiderian membrane intact after aspiration; (F) Membrane elevation creating subantral space; (G) Filling with Bio-Oss® xenograft; (H) Placement of Bio-Gide® resorbable collagen membrane.

Under local anesthesia (2% mepivacaine with 1:100,000 epinephrine), a full-thickness mucoperiosteal flap was elevated through a crestal incision extending from the canine to the second molar, with vertical releases. The lateral sinus wall was exposed (Figure 3A).

A lateral osteotomy created a bony window, revealing the Schneiderian membrane (Figure 3B). The dome-shaped cyst was evident beneath the window (Figure 3C). A small incision was made in the membrane overlying the cyst, and the contents - a viscous, sanguinolent fluid - were aspirated with a syringe (Figure 3D). The Schneiderian membrane remained intact after aspiration, with no perforation (Figure 3E). Because the lesion was managed by aspiration rather than enucleation, no solid cystic tissue was removed, allowing preservation of the Schneiderian membrane and maintenance of sinus physiology. The membrane was then carefully elevated to create a subantral cavity (Figure 3F). The space was filled with ≈2.0 cm³ of particulate xenograft (Bio-Oss®, Geistlich Pharma AG, Wolhusen, Switzerland) (Figure 3G), and the window was covered with a resorbable collagen membrane (Bio-Gide®, Geistlich Pharma AG, Wolhusen, Switzerland) (Figure 3H). Flap closure was achieved with interrupted sutures using Vicryl® 4-0 (Ethicon, Johnson & Johnson, São José dos Campos, Brazil). 

Implant placement

**Figure 4:**
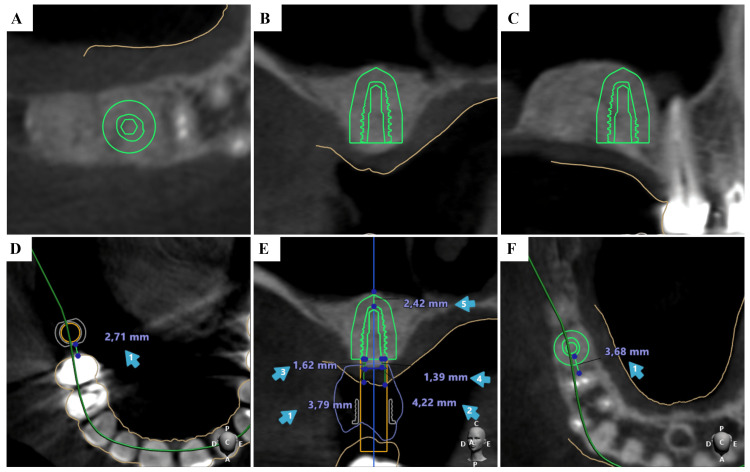
Digital Implant Planning in the #16 Region Using 3D Cone-Beam Computed Tomography (CBCT) Digital implant planning in the #16 region using 3D CBCT reconstruction, illustrating the ideal position in the previously grafted sinus after sinus floor elevation. The highlighted element represents the virtually planned dental implant positioned within the previously grafted maxillary sinus. (A) Axial (occlusal) CBCT view showing implant position and diameter; the brown line indicates soft tissue thickness. (B) Coronal CBCT view showing implant positioning within the augmented sinus. (C) Sagittal CBCT view illustrating the implant in relation to the residual alveolar ridge and grafted bone. (D) Curved multiplanar CBCT reconstruction demonstrating the implant trajectory. (E) Cross-sectional CBCT view with linear measurements of available bone dimensions and safety distances. (F) Axial CBCT view showing the implant position relative to the sinus cavity.

**Figure 5:**
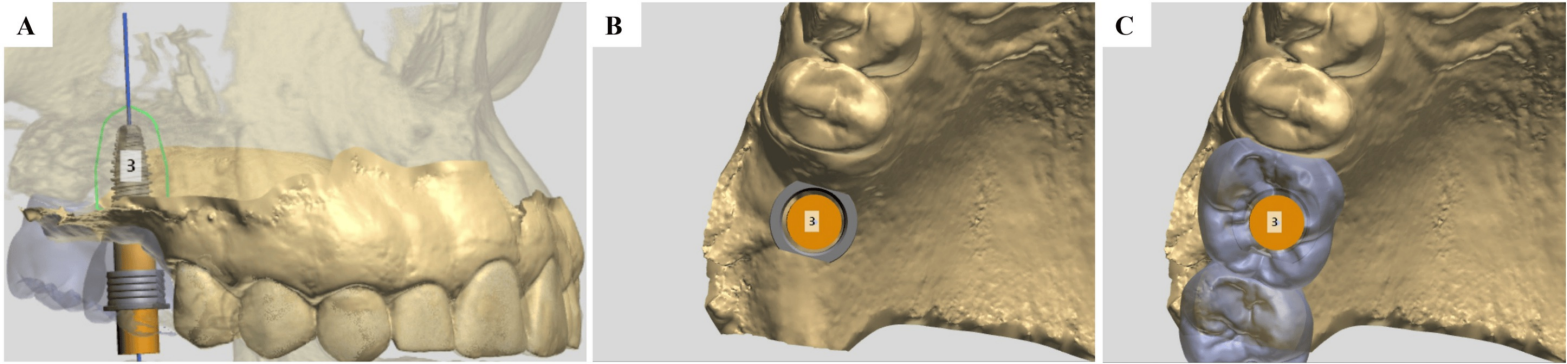
Digital Waxing for 3D-Printed Surgical Guide The image illustrates the digital waxing and virtual design of the 3D-printed surgical guide used for guided implant placement. (A) Cross-sectional cone-beam computed tomography (CBCT) view with superimposed virtual implant planning, illustrating the prosthetically driven implant axis in the #16 region. (B) Occlusal view of the digital implant position relative to the alveolar ridge after sinus augmentation. (C) Digital wax-up of the prosthetic crown used to guide the design of the tooth-supported surgical guide.

Eight months after the SFE, with confirmed complete resolution of the MRC and favorable bone maturation, a digital plan was generated for implant placement in site #16 (Figure 4). A 3D-printed surgical guide was fabricated (Figure 5), and one UNITITE CM Ø4.3 × 8.5 mm implant (S.I.N. Sistema de Implante Nacional S/A, Brazil) was placed, with excellent primary stability.

**Figure 6:**
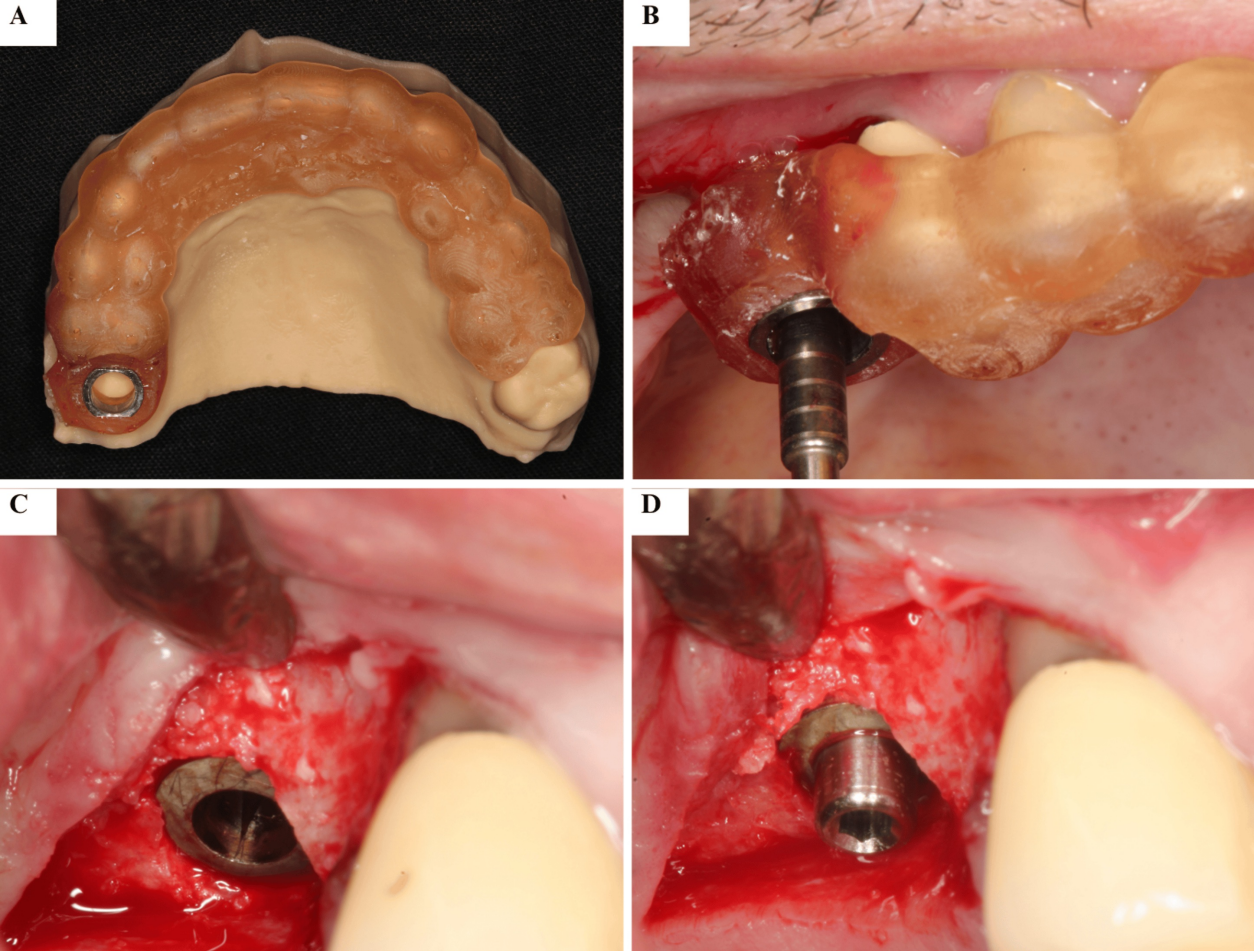
Placement of the Endosseous Implant at Site #16 Placement of the UNITITE CM Ø4.3 × 8.5 mm endosseous implant (S.I.N. Sistema de Implante Nacional S/A, Brazil) in the #16 region using the surgical guide, achieving excellent primary stability. A healing abutment was installed immediately after implant placement. The image shows the placement of the endosseous implant in the #16 region using the surgical guide, with immediate installation of a healing abutment. (A) Surgical guide adapted to the 3D dental model. (B) Surgical guide in position guiding implant installation. (C) Intraoperative appearance after implant placement. (D) Healing abutment installed immediately after implant placement.

Although the implant was positioned with the aid of a 3D-printed surgical guide, flap elevation was required to allow direct visualization of the regenerated sinus floor, controlled harvesting of a trephine core for histologic evaluation, and proper soft tissue management. Guided surgery does not preclude flap elevation, particularly in cases involving previously grafted sites where verification of bone maturation and accurate biopsy collection is necessary. A healing abutment was installed, and closure was achieved (Figure 6).

**Figure 7:**
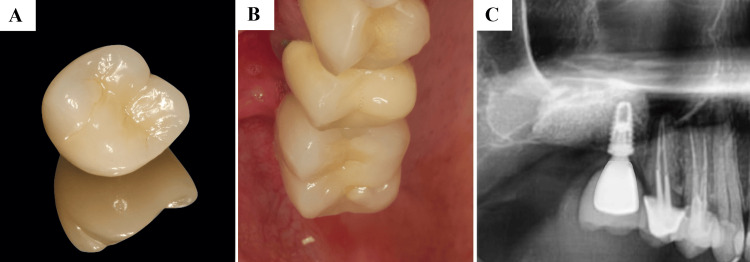
Prosthetic Rehabilitation of the #16 Implant Prosthetic rehabilitation of the #16 implant after the healing period (approximately six months following implant placement). (A)-(C) Clinical and radiographic views showing the definitive crown in function, with adequate peri-implant soft tissue health and proper osseointegration.

Six months later, a screw-retained fixed prosthesis was delivered, completing functional rehabilitation (Figure 7).

Tomographic analysis

CBCT scans were taken immediately after the grafting surgery (T1), eight months postoperatively at implant placement (T2), and six years after bone grafting (T3).

A third CBCT was obtained at the five-year follow-up owing to the initial presence of a large sinus lesion and the aim of documenting long-term graft stability, implant osseointegration, and the absence of cyst recurrence. The scan was performed with a reduced 8 × 8 cm field of view (FOV) and standardized acquisition parameters to minimize radiation exposure while ensuring clinically relevant diagnostic information.

The CBCT model iCat Classic (Imaging Science International, Hatfield, EUA) with exposure factors of 120 kV and 36.12 mAs, with 0.25 mm reconstruction interval and slice thickness was used. Linear and volumetric measurements were performed using 3D Slicer software (version 5.6.1). Semi-automatic segmentation was carried out with threshold-based region growing, followed by manual refinement. RBH, augmented sinus dimensions (height and width), graft volumes, and cyst volumes were calculated in triplicate by the same calibrated operator, and intra-observer reproducibility was confirmed (intraclass correlation coefficient > 0.90).

Density values reported as “Hounsfield Units” (HU) correspond to device-reported CBCT gray scale values calibrated by the manufacturer’s software and do not represent absolute CT-derived HU. This standardization allows for comparative evaluation within the same device and patient across different time points.

Histological analysis

**Figure 8:**
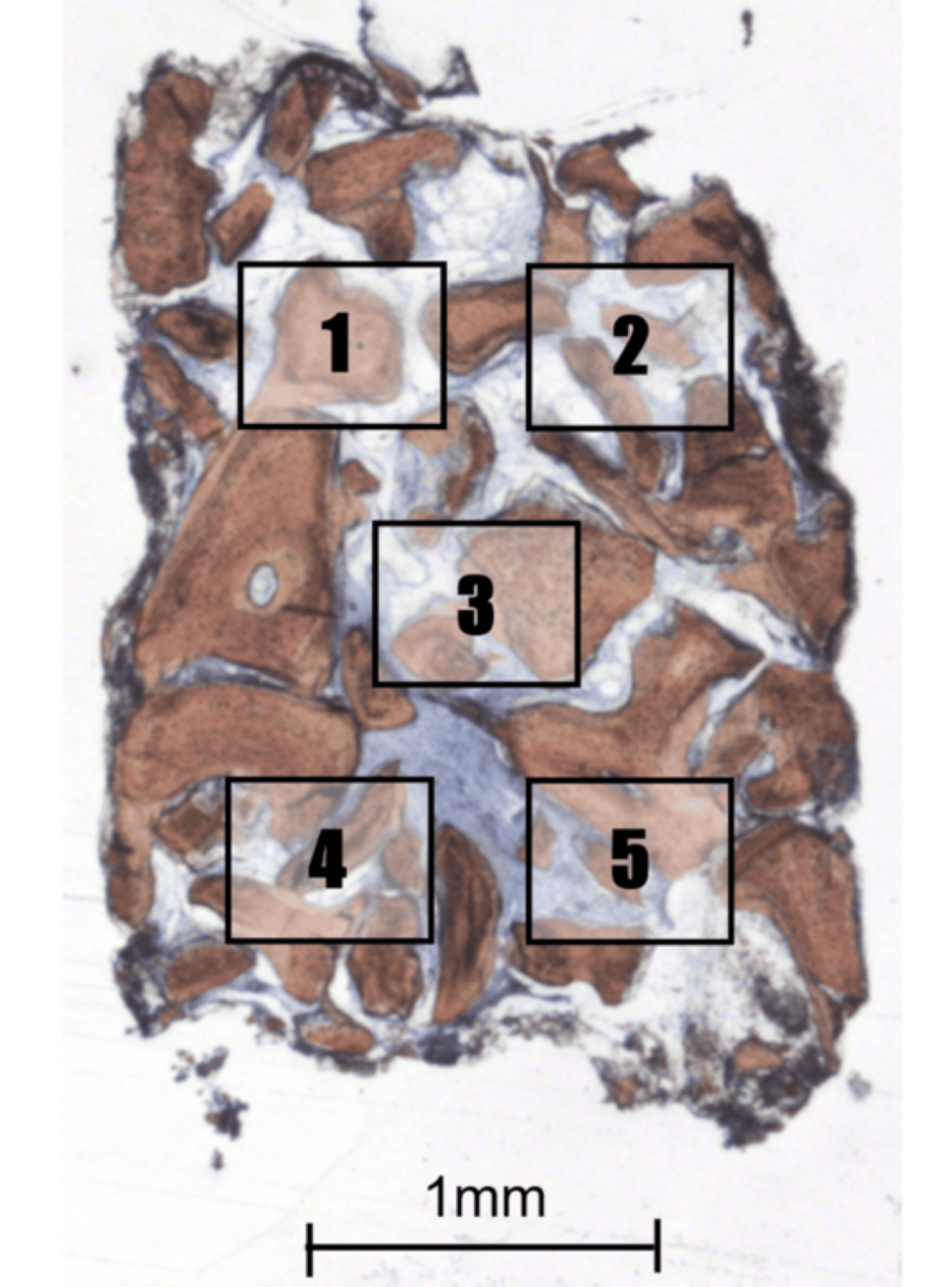
Histological Section of the Biopsy Specimen Histological section of the biopsy specimen stained with Stevenel’s Blue and Alizarin Red (200×). New bone (NB) is observed in close contact with residual graft (RG) particles, with interposed connective tissue (CT) showing good vascularization. A low-magnification photomicrograph (16×) showing the trephine-harvested biopsy specimen, with labels 1-5 indicating the five randomly selected histological fields subsequently analyzed at 200× magnification for histomorphometric point-counting of new bone, residual graft particles, and connective tissue.

At eight months, during implant placement, a bone core biopsy was obtained from the grafted sinus using a 2-mm trephine drill (Figure 8). The specimen was fixed in 10% neutral-buffered formalin, decalcified, processed, and stained with Alizarin Red and Stevenel’s Blue. Histological evaluation assessed the percentages of newly formed bone, residual graft particles, connective tissue, vascularization, and inflammatory infiltrate.

**Figure 9:**
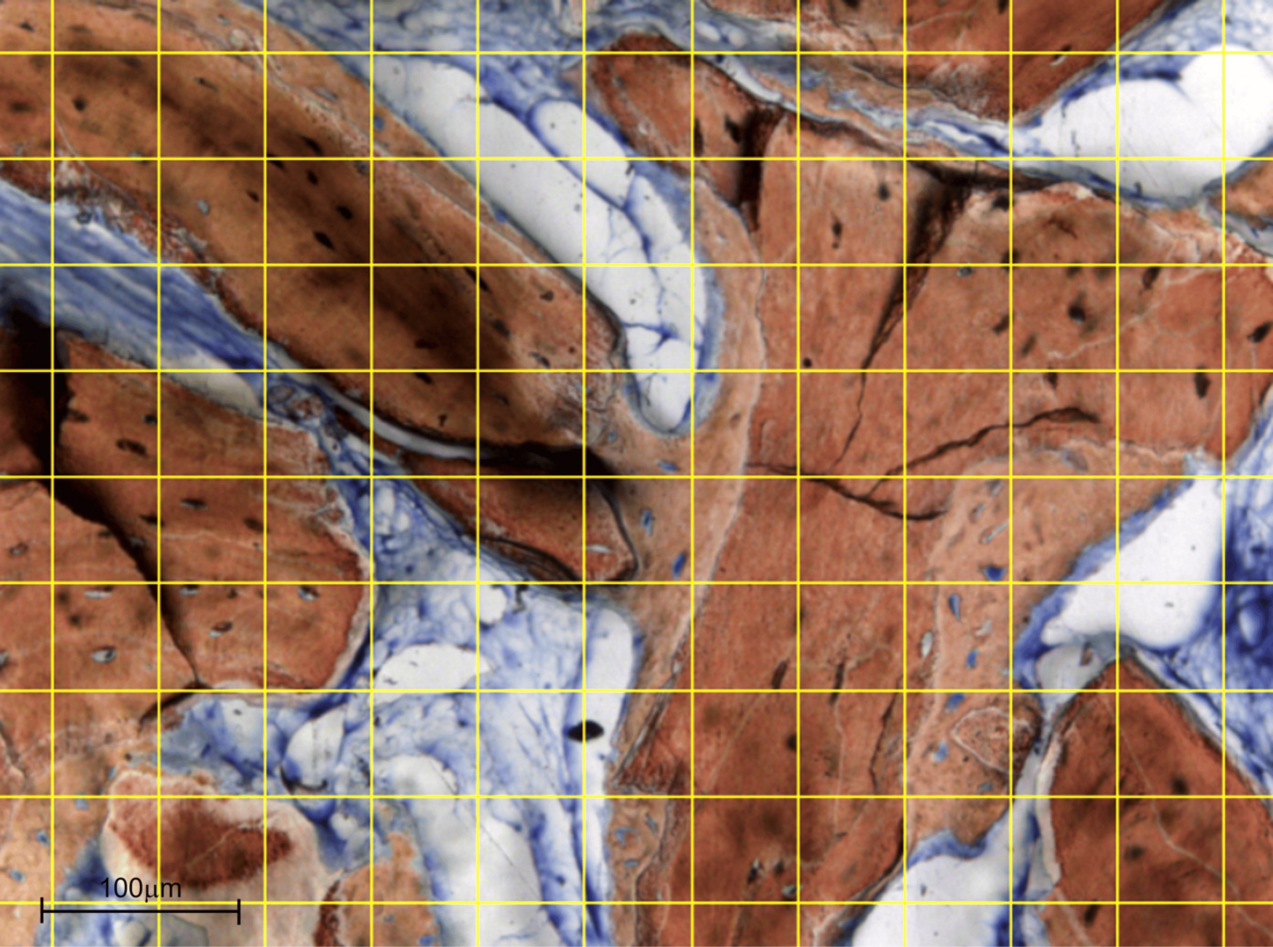
Histomorphometric Analysis of the Biopsy Specimen Histomorphometric grid analysis (200×). A reticulated point grid was superimposed using ImageJ software to quantify the proportions of new bone (NB), residual graft (RG), and connective tissue (CT) (108 intercept points per image).

An Eclipse Ci optical microscope (Nikon Corporation, Tokyo, Japan) equipped with a digital camera (Digital Sight DS-2Mv, Nikon Corporation, Tokyo, Japan) connected to a computer was used to capture photomicrographs for histomorphometric measurements. Digital images were obtained from five randomly selected fields at 200× magnification. For quantitative analysis, a reticulated point grid was superimposed on each image, with 108 intercept points scored per field, using ImageJ software (version 1.34s; National Institutes of Health, Bethesda, MD, USA). Histological structures present at each intersection were classified as new bone, residual graft, or connective tissue, following the point-counting method described by Schroeder and Münzel-Pedrazzoli [17], as shown in Figure 9. The results were expressed as mean ± standard deviation (SD).

Results

Clinical Outcomes

Postoperative healing was uneventful. At all time points, the patient remained asymptomatic, with no infection, sinus complications, or mucosal changes. Peri-implant tissues remained healthy during the entire follow-up. At the six-year evaluation (T3), the implant was stable, functional, and free of complications, with no recurrence of the MRC.

Tomographic Outcomes

**Figure 10:**
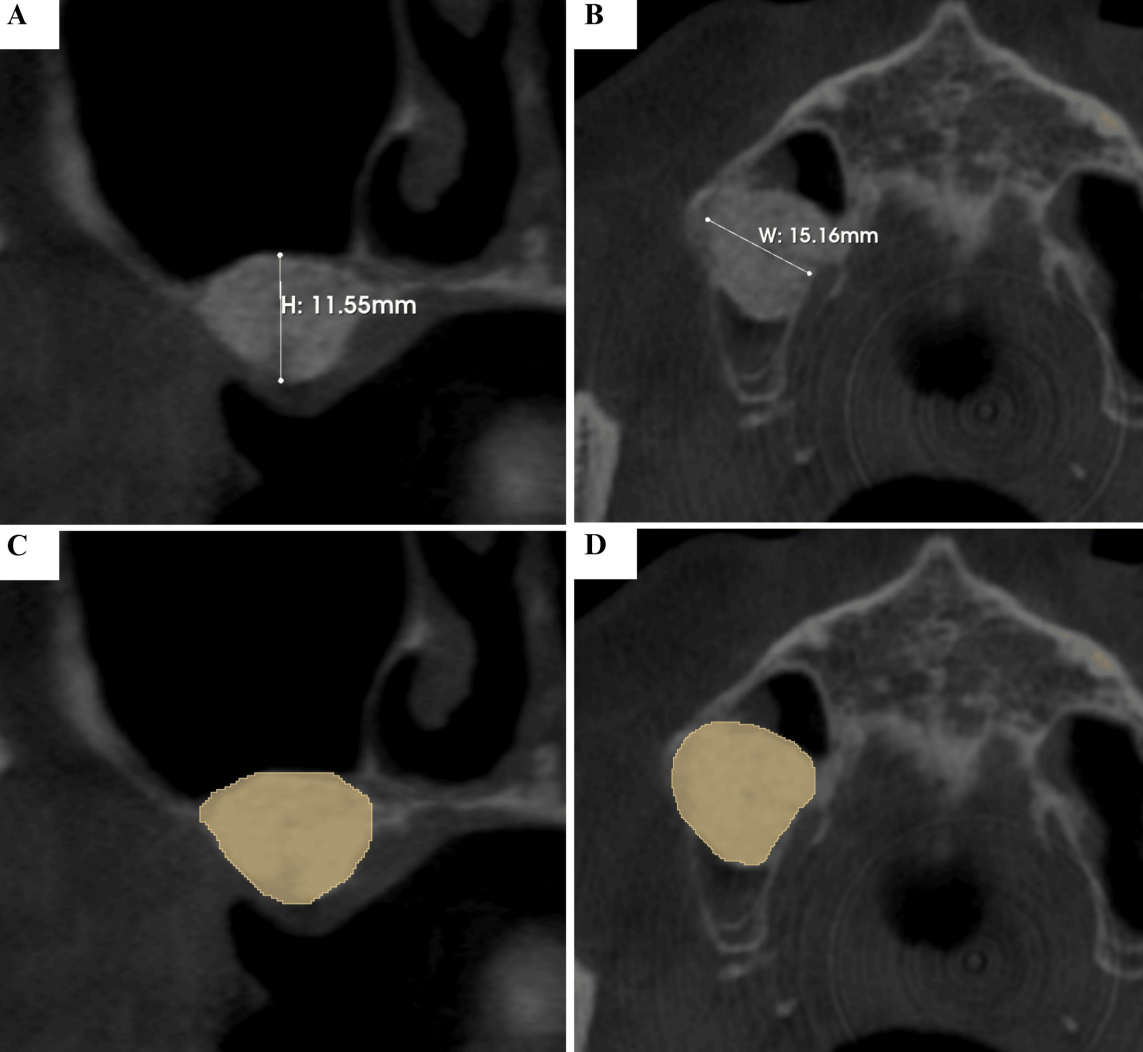
Cone-Beam Computed Tomography (CBCT) Analysis of the Grafted Area at T1 The grafted area at T1 showed a height of 11.55 mm and a width of 15.16 mm. Densitometric analysis revealed an initial mean Hounsfield Unit (HU) value of 297.24 HU, with an overall grafted bone volume of 1978.61 mm³. The highlighted region represents the grafted area within the maxillary sinus, from which linear, volumetric, and CBCT-based densitometric measurements were obtained. (A) Sagittal CBCT slice showing the vertical height of the grafted area (11.55 mm). (B) Axial CBCT slice demonstrating the horizontal width of the grafted area (15.16 mm). (C) Sagittal view with segmentation of the region of interest (ROI) for volumetric and densitometric analysis. (D) Axial view with ROI segmentation used for three-dimensional volume assessment.

Tomographic evaluation demonstrated expected remodeling of the graft. At T1 (one month), the graft volume measured 1,978.61 mm³, with a mean density of 297.24 HU (Figure 10).

**Figure 11:**
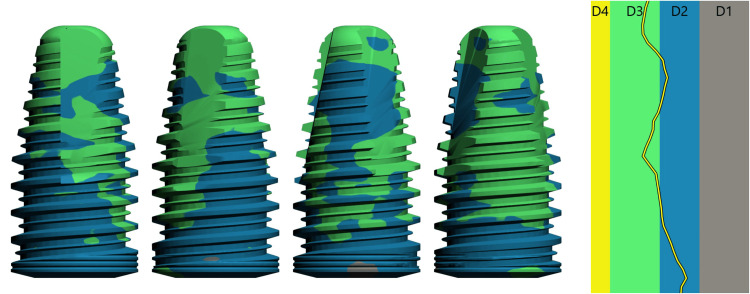
3D Representation of Bone Density in Contact With the Different Areas of the Implant (Bone Types D2 and D3)

The MRC had regressed by 6.10% compared with baseline, measuring 3,067.48 mm³. At T2 (eight months), the graft volume decreased to 1,737.67 mm³, representing a 12.18% reduction compared with T1, while density increased to 690.74 HU. Importantly, the MRC was no longer visible radiographically, confirming complete resolution. At T3 (six years), the graft volume remained stable at 1,508.57 mm³, corresponding to a 23.76% reduction from T1, while density further increased to 785.91 HU, indicating long-term bone maturation and consolidation (Table 1 and Figure 11).

**Table 1:** Tomographic Evaluation T1: one-month postoperative; T2: eight-month postoperative; T3: six-year follow-up; CBCT: cone-beam computed tomography

	CBCT parameters			
	Bone gain			Bone quality
Time	Vertical (mm)	Horizontal (mm)	Volume (mm³)	Density (HU)
T1	11.55	15.16	1978.61	297.24
T2	10.78	13.59	1737.67	690.74
T3	10.58	12.97	1508.57	785.91

**Figure 12:**
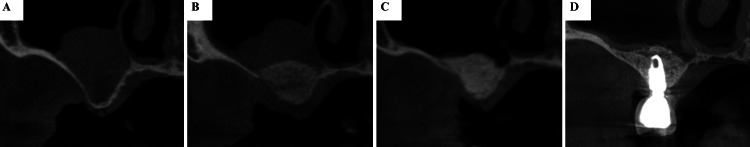
Cone-Beam Computed Tomography (CBCT) Follow-Up of the Grafted Maxillary Sinus CBCT images at different follow-up periods: (A) Pre-operative (T0); (B) One month after sinus floor elevation (T1), showing well-positioned graft material and partial regression of the mucous retention cyst; (C) At eight months (T2), the grafted area exhibited advanced bone maturation and complete radiographic resolution of the cyst, allowing safe implant placement; (D) At the six-year follow-up (T3), the grafted bone demonstrated long-term stability and maturation, with the implant maintaining complete osseointegration and no recurrence of the mucous retention cyst.

The results previously described are shown in Figure 12, which contains CBCT images at the different follow-up periods: T0 (preoperative), T1 (one month after SFE), T2 (eight months postoperative), and T3 (six-year follow-up). 

Histological and Histomorphometric Outcomes

**Figure 13:**
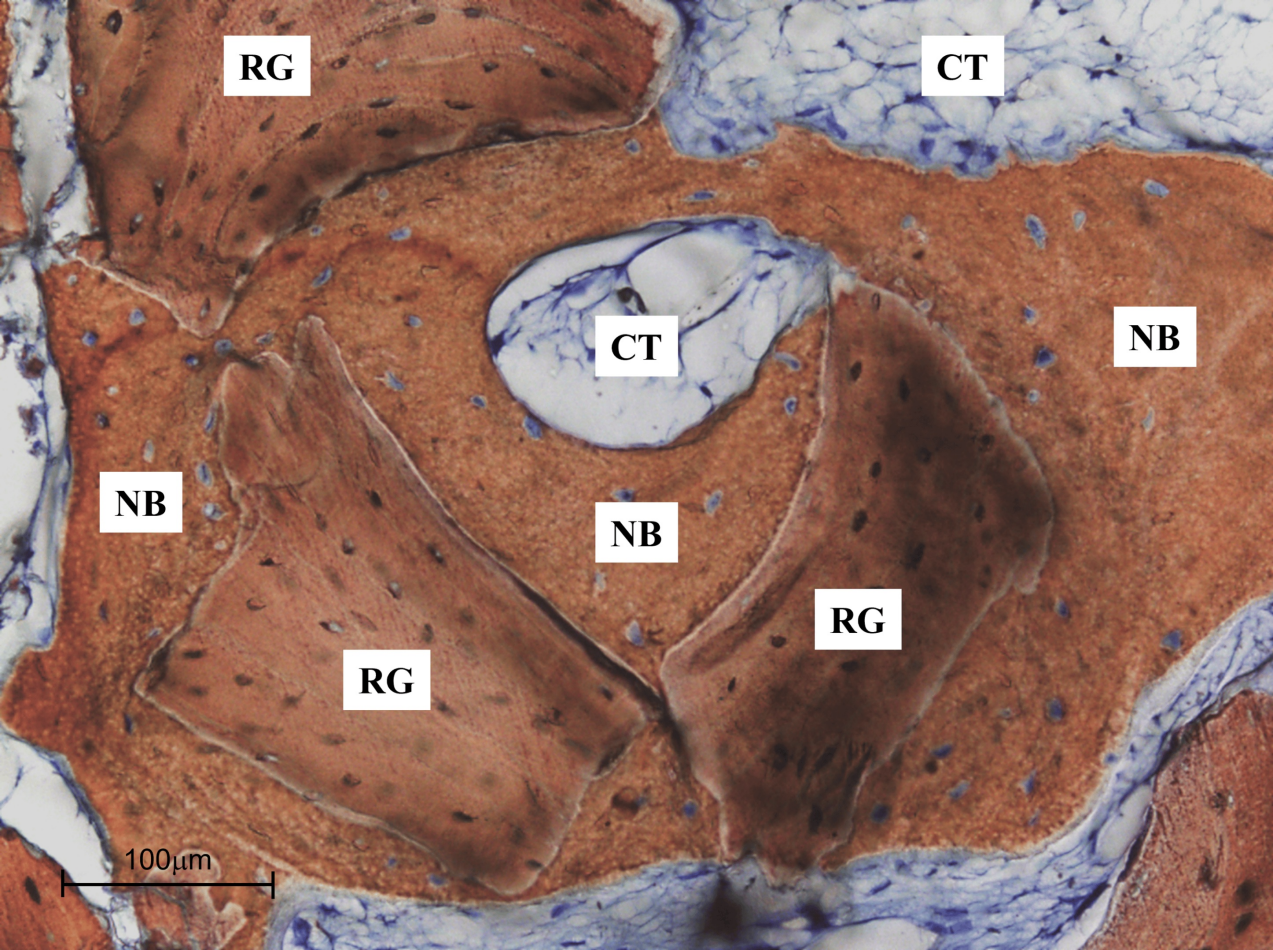
High-Magnification Histological View of New Bone Formation Higher magnification view of new bone formation around graft particles (200×). Osteoblast lining and osteoid deposition are evident, indicating active bone remodeling. NB: new bone; RG: residual graft; CT: connective tissue

Histological analysis of the biopsy obtained at implant placement (eight months) revealed new bone formation in intimate contact with residual xenograft particles. The connective tissue surrounding the graft was well vascularized, with no evidence of inflammatory infiltrate. These findings demonstrated a favorable biological environment and ongoing bone remodeling within the augmented sinus. Histomorphometric evaluation confirmed these observations, showing 25.76% ± 3.15 of new bone, 47.20% ± 3.91 of residual graft particles, and 27.04% ± 3.68 of connective tissue (Table 2 and Figure 13).

**Table 2:** Histomorphometric Evaluation

Histomorphometry (%)			
Area	New bone	Residual graft	Connective tissue
1	20.40	50.0	29.60
2	26.90	46.20	26.90
3	26.80	41.70	31.50
4	28.70	46.30	25.0
5	26.0	51.80	22.20
Mean ± SD	25.76 ± 3.15	47.20 ± 3.91	27.04 ± 3.68

For comparison, a recent study on Bio-Oss® in sinus augmentation reported lower new bone formation (≈17%) and residual graft (≈38%) at a similar healing interval. The higher new bone percentage observed in the present case may reflect the longer healing period (eight months), highlighting the progressive maturation and remodeling of grafted bone over time, and confirming the osteoconductive potential of the xenograft [18].

## Discussion

Management of maxillary sinus MRCs in patients requiring lateral SFE has evolved from routine pre-grafting removal to more selective, minimally invasive strategies tailored to cyst size, membrane integrity, and surgical access. Early reports and reviews established that many MRCs are incidental, often asymptomatic lesions with variable natural history, supporting an individualized approach rather than systematic eradication in every case [3-5]. Against this background, contemporary implant literature confirms that SFE can achieve high long-term survival even in challenging posterior maxillae, with classical systematic reviews reporting robust outcomes at ≥5 years [1] and favorable performance when RBH is <4 mm, treated via the lateral window technique [2].

Within this framework, three broad strategies have been described when an MRC is encountered at the time of SFE: (i) simultaneous micro-invasive enucleation through the lateral antrostomy when the lesion is accessible and the Schneiderian membrane can be preserved [8]; (ii) simple aspiration/decompression of cystic contents to restore working space and allow safe elevation without perforation [13]; and (iii) retention/observation of small, non-obstructive lesions when adequate elevation can be achieved without manipulating the cyst [12,15,17]. Cohort studies and case series document that both removal and retention, when judiciously selected, can yield predictable graft consolidation and implant survival [9-11,15]. Notably, histology obtained during simultaneous removal has shown vital bone interdigitating with xenograft particles and minimal inflammation by six months, aligning with the biopsy in the present case [11].

Randomized and observational data further nuance decision-making. A randomized comparison of immediate versus delayed SFE after pseudocyst removal found similar implant-level outcomes when membrane integrity and sinus drainage were respected, supporting a one-stage pathway in carefully selected scenarios [14]. A retrospective cohort comparing removal versus retention reported no clinically meaningful differences in graft maturation or implant performance, provided that the lesion did not obstruct ostiomeatal complex function, and the membrane remained intact [15]. Algorithms proposed in broader series similarly recommend cyst-specific planning (size, location, and membrane status), prioritizing atraumatic elevation and maintenance of sinus physiology [12].

The present case mirrors these principles: aspiration through the lateral window re-established working space without perforation, enabling tension-free membrane elevation and stable graft placement. Topographically, the grafted volume showed the expected early contraction from T1 to T2, with continued densification and long-term stability at six years - results consistent with typical remodeling behavior after lateral SFE and with the high survival estimates reported in long-term series and meta-analyses [4,19,20]. When inadvertent membrane injury occurs in cystic sinuses, biologic membranes such as platelet-rich fibrin have been used to manage perforations and proceed safely, broadening the therapeutic window in selected cases [16]. One-stage aspiration/enucleation with simultaneous SFE, and even implant placement, has also been reported with favorable short-term results in well-indicated patients [8,13,20]. It should also be acknowledged that no histopathologic diagnosis was obtained in the present case. This was not an omission, but rather an inherent consequence of the chosen minimally invasive strategy, as the lesion was managed by aspiration/decompression rather than enucleation, and no solid cystic lining was available for submission. In asymptomatic, dome-shaped lesions with classic radiographic features of MRCs, several authors have emphasized that diagnosis is predominantly radiologic and clinical, and that routine excision solely for histologic confirmation is not mandatory when malignancy or aggressive pathology is not suspected [12,15,17]. Importantly, preservation of the Schneiderian membrane and maintenance of sinus physiology are considered higher priorities than tissue retrieval in such scenarios, particularly when the lesion does not compromise sinus drainage or surgical access. The long-term clinical and tomographic stability observed in the present report, with no evidence of recurrence or sinus dysfunction, further supports the adequacy of this conservative, diagnosis-driven approach.

To enhance methodological consistency and permit a more objective long-term interpretation of tomographic and histomorphometric outcomes, grafting in the present case relied exclusively on xenograft particles combined with a collagen membrane. The deliberate avoidance of autologous blood derivatives or composite grafts aimed to minimize biologic variability that could confound the assessment of density changes, volumetric remodeling, and new bone formation over extended follow-up, thereby allowing a clearer depiction of the intrinsic remodeling behavior typically observed after lateral SFE [1,4].

Finally, current evidence suggests that the mere presence of an MRC is not an independent risk factor for implant failure after SFE when cases are properly selected and executed [20]. Taken together, contemporary data support a pragmatic, minimally invasive, and anatomy-driven approach: remove when easy and safe; aspirate to regain space when needed; or retain and observe if the lesion is small and non-obstructive - always prioritizing membrane integrity and sinus ventilation. The clinical, tomographic, and histologic outcomes in this report align closely with that evidence-based pathway.

## Conclusions

This case demonstrates that simultaneous management of a large MRC during lateral SFE is a feasible and predictable approach in the atrophic posterior maxilla. The single-stage procedure allowed for effective cyst aspiration, successful grafting with a particulate xenograft and collagen membrane, and subsequent implant placement with guided precision. Five-year clinical and radiographic follow-up confirmed stable grafted bone volume, complete osseointegration, and no recurrence of the sinus lesion. These findings support the consideration of a combined, single-stage approach for the management of MRCs in patients requiring sinus augmentation, potentially reducing treatment time and surgical morbidity while achieving long-term implant success. One of the inherent limitations of this study is the potential variability in CBCT-based measurements. This was mitigated by having all linear, volumetric, and densitometric analyses performed by a single calibrated operator, ensuring consistency and minimizing inter-operator discrepancies.
